# Fetal Intraventricular Hemorrhage Due to Antiphospholipid Syndrome: A Case Report

**DOI:** 10.3389/fped.2020.622597

**Published:** 2021-02-05

**Authors:** M. C. Bouw, S. W. A. Nij Bijvank, J. W. Bouwhuis, G. van Wezel-Meijler

**Affiliations:** ^1^Department of Neonatology, Isala Women and Children's Hospital, Zwolle, Netherlands; ^2^Department of Obstetrics and Gynecology, Isala Women and Children's Hospital, Zwolle, Netherlands; ^3^Department of Internal Medicine, Isala Hospital, Zwolle, Netherlands

**Keywords:** antiphospholipid syndrome, obstetric antiphospholipid syndrome, antiphospholipid antibodies, prematurity, lupus anticoagulant, case report, fetal intraventricular hemorrhage

## Abstract

Obstetric Antiphospholipid Syndrome (OAPS) is an autoimmune disease characterized by certain pregnancy complications in association with persistent antiphospholipid antibodies. These antibodies are generally known for their prothrombotic characteristics and may affect mother and fetus during the entire pregnancy. The clinical criteria for OAPS, including recurrent fetal loss, intra-uterine growth restriction and premature birth due to severe preeclampsia, all suggest uteroplacental vascular insufficiency. Although rare, thrombotic complications have been described in neonates born to mothers with OAPS, mainly ischemic stroke. We report on the first case of extensive fetal intraventricular *hemorrhage* related to OAPS. We share our diagnostic search and analysis for this unusual antenatal event, including cranial ultrasound findings and postmortem MRI images. We will also present a short review of the etiology and prognosis of antenatal intraventricular hemorrhage. We suggest that women with severe or early preeclampsia and/or a history of pregnancy loss should be evaluated for OAPS and carefully monitored throughout pregnancy. Further, we advise to test mothers for OAPS in the case of idiopathic fetal hemorrhage.

## Introduction

Antiphospholipid syndrome (APS) is an acquired autoimmune disorder characterized by recurrent venous and/or arterial thrombosis, that can occur in virtually any organ, in the presence of elevated antiphospholipid antibodies (Table 1) (1, 2). APS in pregnant women is sometimes referred to as “obstetrical” APS (OAPS) (3, 4). These women present with complications such as recurrent fetal loss, premature birth, intra-uterine growth restriction, and severe preeclampsia. Prematurity occurs in almost half of the women with OAPS, because of early termination of pregnancy due to deteriorating maternal condition in severe preeclampsia. In this case report, we show how OAPS, in a previously healthy appearing pregnant woman, can result in severe fetal intraventricular hemorrhage.

**Table 1:** Revised Sapporo criteria for APS.

**Clinical criteria**	
Vascular thrombosis	One or more episodes of arterial, venous, or small-vessel thrombosis in any tissue or organ^¥^
Pregnancy morbidity	A. One or more unexplained deaths of a morphologically normal fetus ≥ 10 weeks of gestation or beyond
	B. One or more premature births of a morphologically normal neonate < 34th week of gestation, owing to: i severe preeclampsia or eclampsia defined according to standard definitions ii recognized features of placental insufficiency^*^
	C. ≥ three unexplained consecutive spontaneous abortions ≤ 10 weeks of gestation after ruling out any maternal anatomic or hormonal abnormalities and paternal and maternal chromosomal causes
**Laboratory criteria**	
Lupus anticoagulant present in plasma, in medium or high titer, on ≥ 2 occasions at least 12 weeks apart^$^	
Anticardiolipin antibody of IgG or IgM isotype in serum or plasma, on ≥ 2 occasions at least 12 weeks apart^#^	
Anti-β2 glycoprotein-I antibody of IgG and/or IgM isotype in serum or plasma, present on ≥ 2 occasions at least 12 weeks apart^#^	

*To diagnose APS, at least one laboratory and one clinical criterion should be met*.

¥ *Thrombosis must be confirmed by appropriate imaging or histologically. For histopathologic confirmation, thrombosis should be present without significant evidence of inflammation in the vessel wall*.

* Generally accepted features of placental insufficiency include: *   a) abnormal or non-reassuring fetal surveillance test(s), e.g., a non-reactive non-stress test, suggestive of fetal hypoxemia*,   b) abnormal Doppler flow velocimetry waveform analysis suggestive of fetal hypoxemia,   c) oligohydramnios, for example, an amniotic fluid index of 5 cm or less, or *   d) a postnatal birth weight less than the 10^th^ percentile for the gestational age*.

$ *according to the guidelines of the International Society on Thrombosis and Hemostasis*.

# *measured by a standardized enzyme linked-immunoabsorbend assay (ELISA), according to recommended procedures*.

Recognition and understanding of APS has taken several decades (5, 6). In the 1980s, Dr. Hughes and his team first described the link of repeated thrombosis, neurological abnormalities and recurrent pregnancy loss with lupus anticoagulant and anticardiolipin antibodies, directed against phospholipids on plasma membranes, and referred to it as “Hughes Syndrome.” Through the years, knowledge of APS has further evolved, including the identification of a third antibody, antiβ2gycloprotein-1, eventually converting the name to “antiphospholipid (antibody) syndrome” (6).

However, our understanding of the pathogenetic mechanisms of the disease and heterogeneous clinical expression are not yet fully understood and the clinical definition of APS has repeatedly changed. To attain more uniformity worldwide, the original classification criteria for APS were formulated in 1998 in Sapporo, Japan, the so-called “Sapporo criteria” (1). Next, in 2006, at the Eleventh International Congress on Antiphospholipid Antibodies in Sydney, Australia, several adjustments were formulated, mainly more precisely defined laboratory cut-off values and standardization. This is now referred to as the “revised Sapporo criteria” (Table 1) (2). Some experts suggest including several frequently observed “minor criteria” such as hemolytic anemia, thrombocytopenia or livedo reticularis (as was the case in our case), but these are not yet included in the classification criteria (1, 2, 4).

## Case

At 8 weeks' gestation, a 28-year-old woman was referred to Internal Medicine with fatigue, arthralgia and mottled blue skin discoloration on the pretibial surfaces of both legs. Other than this rash, physical examination was unremarkable. Obstetrical history reported two uncomplicated pregnancies (same partner) with full-term birth of two healthy sons with normal birth weights, respectively seven and five years earlier. During these pregnancies, the woman had received iron and vitamin supplements.

At consultation, the patient indeed had mild microcytic anemia and a low level of vitamin D. Also, an elevated erythrocyte sedimentation rate (ESR) was noted (Table 2). Again, oral vitamin D and iron were prescribed. The increased ESR was presumed to be pregnancy-related (7). At follow-up 2 months later, she experienced less pain and fatigue, and serum vitamin D and hemoglobin levels were within normal range. However, ESR was still considerably elevated (Table 2). Assuming that the advanced pregnancy was the cause hereof, it was decided to postpone further analysis until after delivery.

**Table 2:** Laboratory results mother.

	**8 week's** **gestation**	**19 week's** **gestation**	**After birth**	**Reference range**
Erythrocyte sedimentation rate	45	51	106	1–20 mm/h
Hemoglobin	10.0	11.3	10.6	> 12.0 g/dl^*^
Mean corpuscular volume	78	82	91	80–100 fL
White blood count	-	4.2	4.1	6.0–14.0 × 10^9^/L^#^
Thrombocytes	-	198	213	150–400 × 10^9^/L
C-reactive protein	3	18	7	< 3 mg/L
25-OH-vit-D	46.0	110.8	137.5	> 50 nmol/L
Complement 3	-	-	80	85–180 mg/dl
Complement 4	-	-	7	15–50 mg/dl
Haptoglobin	-	-	2.0	0.35–2.2 g/L

* *Hb in pregnancy: first trimester > 11.6 g/dl, second trimester > 9.7 g/dl*.

# *WBC in pregnancy is slightly higher than in non-pregnancy*.

The patient was seen for obstetric ultrasound examination in our hospital at 19^+3^ and again at 22^+1^ weeks' gestation because of echogenic fetal bowel at routine second-trimester ultrasound examination. During repeated advanced ultrasound scanning, including the brain, no fetal abnormalities were noticed.

At 26^+1^ weeks' gestation the patient presented with abdominal pain and physical examination revealed serious hypertension (180/115 mmHg). Laboratory analysis showed hemolysis, elevated liver enzymes, thrombocytopenia (HELLP syndrome), and significant proteinuria (0,65 g/L) indicating severe preeclampsia. Because cardiotocography showed an abnormal fetal heart rate pattern delivery by Cesarean Section was performed.

### The Neonate

**Figure 1:**
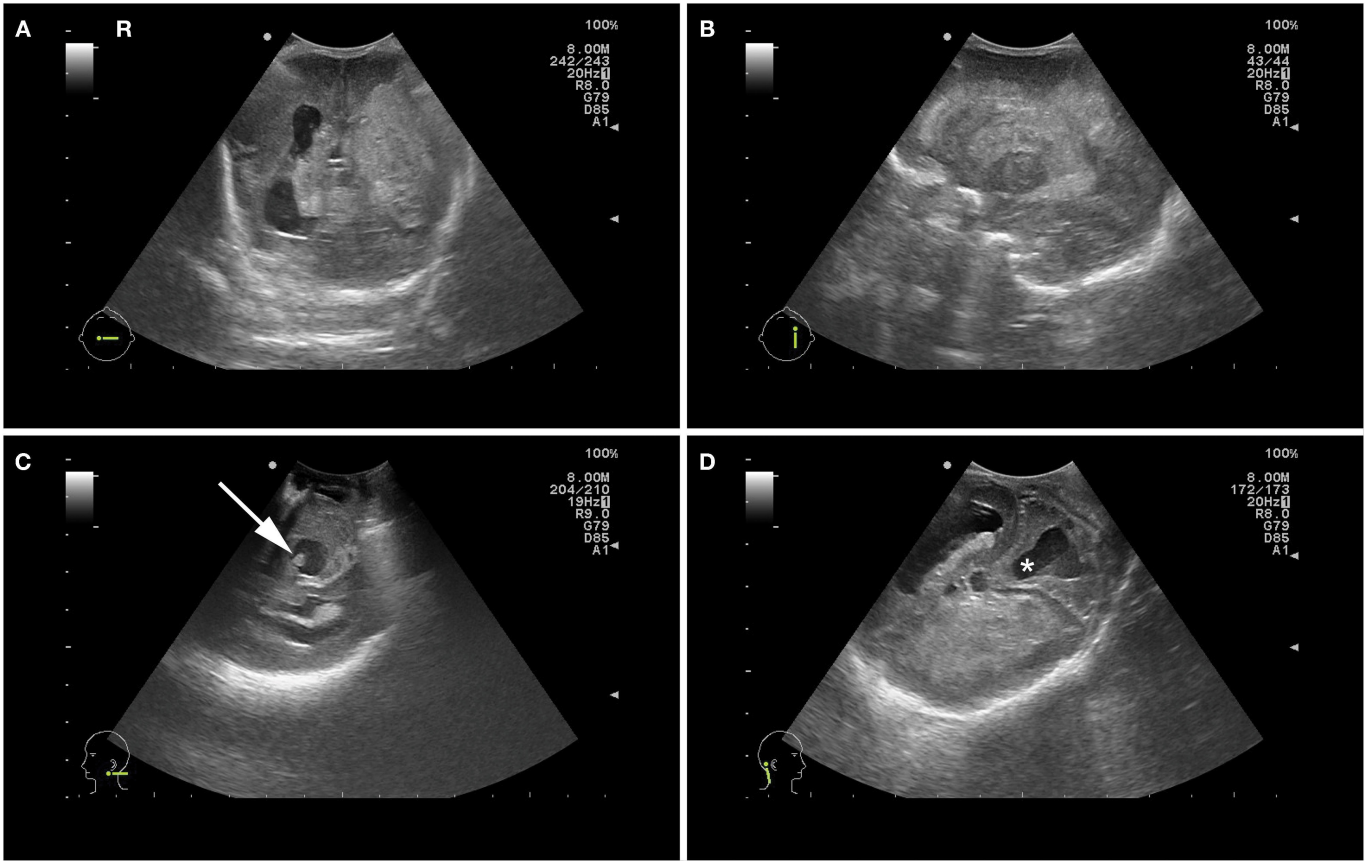
Postnatal cranial ultrasound images showing bilateral intraventricular hemorrhage with extensive hemorrhagic infarction of the left hemisphere and ventricular dilatation in coronal view **(A)** and parasagittal left plane view **(B)**, blood clot in the 4^th^ ventricle **(C)** and dilatation of the 4^th^ ventricle (*) with dysplastic cerebellar hemispheres **(D)**.

A vigorous female neonate was born, with a birth weight of 540 grams (−1,65 SD). After stabilization, cranial ultrasound examination was performed within 3 h after birth (Figure 1). This showed extensive bilateral intraventricular hemorrhage (IVH) with profound parenchymal involvement due to venous infarction of the left hemisphere, also referred to as (periventricular hemorrhagic infarction). Resorbing blood clots were present in the third and fourth ventricle with severe bilateral ventricular dilatation. In addition, cerebellar destruction was seen, probably resulting from the toxic effect of hemosiderin deposits due to hemolysis of the blood clot in the fourth ventricle (8, 9). Doppler ultrasonography showed normal flow velocities in the cerebral venous system, making a cerebral venous sinus thrombosis as the primary cause of the IVH unlikely.

Considering the extensive cerebral injury combined with the extreme prematurity and growth restriction, redirection of care was proposed. The girl succumbed 2 days later.

Because of the uncommon presentation of severe fetal intraventricular hemorrhage, we aimed to find an explanation to why and when this bleeding had occurred.

**Figure 2:**
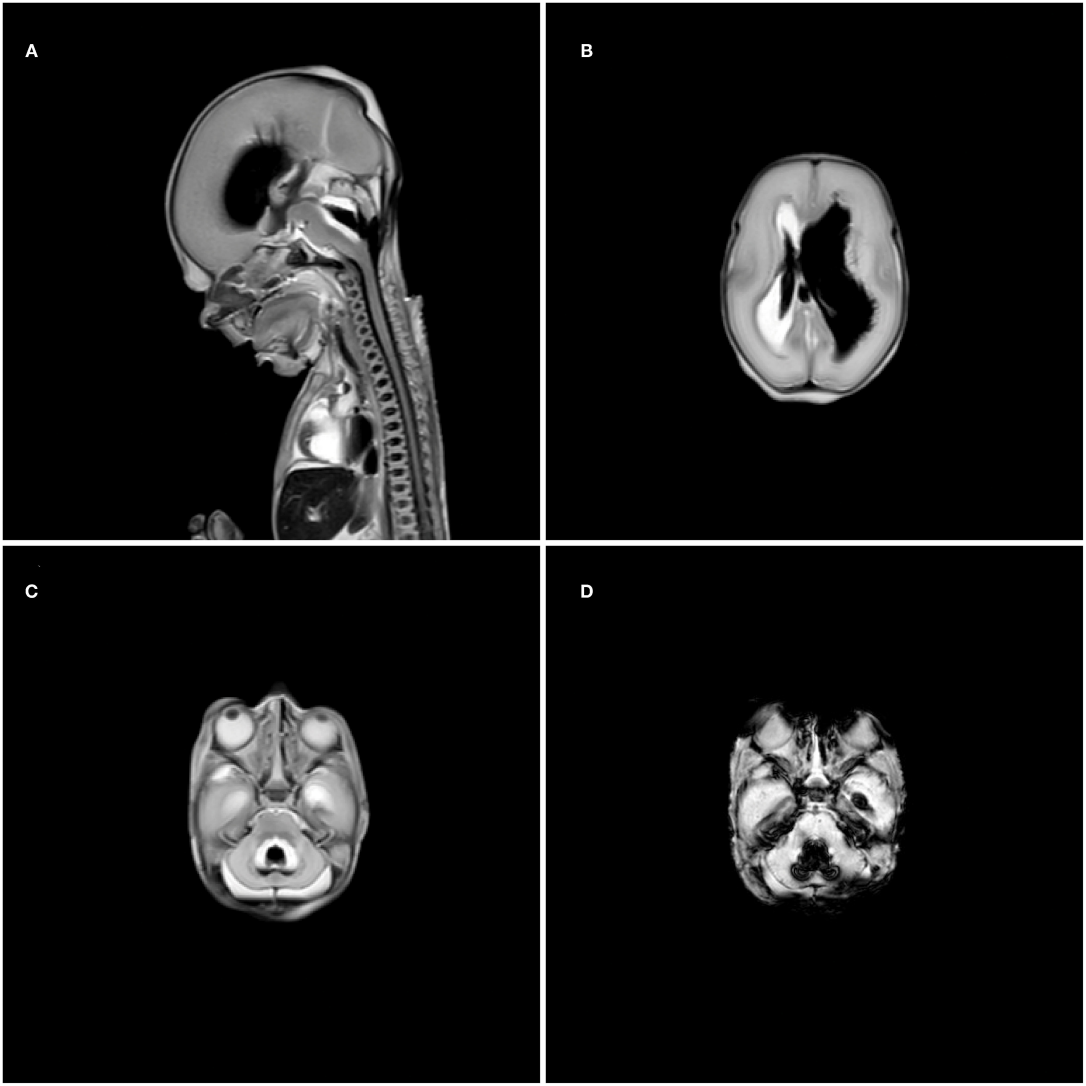
Postmortem magnetic resonance imaging showing extensive ventricular dilatation and blood in the 4^th^ ventricle in T2-weighted midsagittal plane **(A)**, intraventricular blood in the right ventricle and ventricular dilatation of the left ventricle in T2-weighted axial plane **(B)**, blood in the 4^th^ ventricle with cerebellar dysplasia in T2-weighted axial plane **(C)** and susceptibility weighted image axial plane **(D)** of the posterior fossa.

The parents did not consent to autopsy in view of their Islamic belief but agreed upon postmortem total body MRI to look for possible other hemorrhages or other clues. MRI confirmed the ultrasound findings of hemorrhage originating from the germinal matrix of the ventricle and did not show any other sites of hemorrhage nor abnormalities otherwise (Figure 2). The prenatal ultrasound scans were re-examined, indeed showing a normal ventricular system without hemorrhage or dilatation at 22 ^+1^ weeks' gestation. We presume that the IVH in our case must have occurred antenatally considering the fact that extensive brain abnormalities were already present directly after birth. More precisely, we estimate it to have taken place somewhere between 7 and 14 days as cerebellar destruction by hemosiderin takes some time to evolve while cystic degeneration of periventricular hemorrhagic infarction, of which no signs were present yet at birth, develops over approximately 14 days.

Neonatal blood was analyzed (Table 3). Coagulation tests were within the normal range for prematurity. Because platelets were moderately decreased, neonatal and parental blood was analyzed for neonatal alloimmune thrombocytopenia (NAIT). HPA-alloantibody detection by MAIPA en PIFT, both with an HPA-typed donor panel and with paternal platelets, were negative. In addition, no free platelet-associated or free circulating platelet autoantibodies were detected, using a glycoprotein-specific (direct MAIPA) assay, making maternal ITP unlikely (13). Fortuitously, this analysis revealed an elevated thrombopoietin (TPO), not considered attributable to the moderate degree of thrombocytopenia. Congenital amegakaryocytic thrombocytopenia (CAMT), a rare inherited bone marrow failure syndrome, due to mutations coding for the TPO receptor was ruled out by microscopic examination of a peripheral blood smear. Remarkable was the unusual increased number of nucleated red blood cells (NRBC's), representing immature erythrocyte precursors in the blood smear (14). Otherwise, the blood smear showed no significant abnormalities; platelet morphology was normal. Genetic testing for COL4A1 and COL4A2 mutations was negative (15).

**Table 3:** Laboratory results neonate.

		**Reference value**
Hemoglobin	10.0	12.7–20.4 g/dl^*^
White blood count	4.0	3.04–31.0 × 10^9^/L^*^
Thrombocytes	65	> 114 × 10^9^/L^*^
C-reactive protein	<1	<1 mg/L
NRBC	27.7	≤ 6.4 × 10^9^/L$
PT	1.6	12.9–28.5s^#^
APTT	63	53.7–139.3s^#^
TPO	608	4–90 pg/ml

* *Reference range for neonates GA < 28 weeks (10)*.

$ *Reference value for neonates GA < 28 weeks on day 1 (11)*.

# *Reference range for neonates GA < 28 weeks (12)*.

Histopathologic examination of the placenta showed a normal umbilical cord and no signs of infection. Most remarkable was the macroscopic finding of extensive placental infarction of the placental bed, occupying 50% of the maternal side of the placenta; microscopically the placenta showed advanced villous maturation and distal villous hypoplasia. These findings are referred to as maternal vascular malperfusion and is considered characteristic in placentae from patients with antiphospholipid antibodies.

### The Mother

Maternal screening for toxoplasmosis, CMV, HIV, HSV, syphilis, and hepatitis A, B, and E was negative. Therefore, an infectious cause of the fetal IVH seems unlikely. Platelet count before and after the HELPP syndrome, were normal (Table 2). The woman did not use other drugs than the medication prescribed in the first trimester and history taking of trauma was negative. Family history was negative for coagulation disorders or auto-immune disease.

Two months after delivery, the mother was re-evaluated at Internal Medicine and referred to a dermatologist. Here, the persistent rash on the legs was recognized as “livedo reticularis,” which is known to be associated with underlying systemic disease, such as systemic lupus erythematosus. Biopsy was not performed because of the absence of an epidermal component. This finding supports our hypothesis of an underlying maternal autoimmune disease, presumably APS. Laboratory analysis showed a persistent elevated ESR and antinuclear antibodies (ANA) and anti-ENA/CTD screening was positive for anti-double-stranded DNA (dsDNA) antibodies and lupus anticoagulant. Anticardiolipin and antiβ2gycloprotein-1 antibodies were negative, however. Other antibodies including anti-Sm and anti-RNP were also negative. Additionally, both complement C3 and C4 were reduced and Coombs test was positive in absence of hemolytic anemia; both indicating formation of excessive immune complexes in APS and SLE (16) (Table 2). However, no cytopenia or proteinuria was present. Despite of this, the mother did not (yet) meet the clinical criteria for a concomitant diagnosis of systemic lupus erythematosus.

Considering these laboratory abnormalities in combination with the premature birth <34 weeks' gestation due to severe preeclampsia and placental insufficiency, the mother meets the clinical criteria for OAPS (Table 1).

A few months later, when diagnosis of OAPS was officially made because of persistently elevated lupus anticoagulant more than 12 weeks apart the mother pragmatically started with low-dose aspirin and hydroxychloroquine as she wished to become pregnant again. Almost 2 years later, she gave birth to a healthy boy, delivered at 37 week's gestation with a birth weight of 3,452 g. The parents showed gratitude for our persevering quest leading to new (born) life.

## Discussion

Several case reports have described neonates with antiphospholipid antibodies associated complications, mainly ischemic stroke (17–22). Antenatal or fetal hemorrhage in relation to OAPS, as in our case, has, to the best of our knowledge, not been reported.

### Fetal Intraventricular Hemorrhage

Germinal matrix intraventricular hemorrhage (IVH) is a well-known and relatively frequently occurring condition in very preterm infants (23). Intracranial hemorrhage, including IVH is however rare in the fetus (24). The true incidence is difficult to assess; studies are mainly based on case reports. In a case study performed in Italy, wherein 6,641 healthy pregnant women had routine prenatal ultrasounds at 20 and 32 weeks' gestation, only 6 cases of fetal intracranial hemorrhage (FIH) were detected (0.9/1,000) (25). Likewise, Elchalal et al. found 33 cases of FIH, most of them classified as IVH, out of a total of 71,023 pregnancies (0.46/1,000) in women undergoing prenatal ultrasound examinations in Israel (26). Both authors state that this may be an overestimation considering the fact that these studies were done in tertiary obstetrical centers. On the other hand, cases may remain undiagnosed, as FIH can either lead to intra-uterine demise or may be asymptomatic or may only present later in infancy or childhood with unexpected neurodevelopmental impairment.

FIH may occur in association with various maternal and fetal conditions, but in most cases the cause remains unidentified. In the case study of Elchalal et al. maternal or fetal conditions were identified in only 45% of FIHs (26). Correspondingly, Ghi et al. could detect a probable underlying etiology in only seven out of 16 cases (43%) (27). These were mainly fetal hematological disorders and maternal trauma (27–29).

Fetal conditions associated with FIH are predominantly those with hemorrhagic diathesis, such as inherited coagulation disorders or neonatal alloimmune thrombocytopenia (24, 27). In addition, only quite recently, two mutations, COL4A1 and COL4A2, impairing formation of collagen proteins in (cerebral) vascular basement membrane have been associated with FIH (23). Furthermore, twin–twin transfusion, demise of a co-twin or fetomaternal hemorrhage have been associated with FIH (24). Maternal risk factors include abdominal trauma, amniocentesis, anti-coagulant medication or drugs such as cocaine (28, 29). Viral or bacterial infections, including Rubella, Cytomegalovirus, Herpes virus and CMV, are also rare risk factors for the occurrence of FIH. Systemic diseases such as systemic lupus erythematosus or SSA/SSB antibody positivity or auto-immune thrombocytopenia have been described in relation to fetal hemorrhage (29).

Because of the unusual occurrence of FIH, data on outcome are limited. In addition, different underlying causal mechanisms will also play a role in neonatal prognosis. When focusing on the prognosis of severe fetal IVH with periventricular hemorrhagic infarction, as in our case, a review of the literature by Ghi et al. reported intra-uterine death in 12 out of 32 cases, while three couples decided to terminate the pregnancy. Of the remaining 17 cases resulting in life births, seven infants had postnatal follow-up data available. Of these seven infants, four were alive at mean age of 11.6 months of whom three were classified as having severe developmental delay and only one as neurologically normal (26). Vergani et al. presented a large series of 39 cases with FIH showing that IVH with parenchymal involvement resulted in fetal or neonatal death or severe neurological deficits in 92% (12/13) of the cases (25).

### In Retrospect: Chronic Fetal Hypoxia

In our case, many findings represent chronic fetal hypoxia in retrospect. First of all, the remarkable high TPO level in the neonate. Moderately raised levels of TPO are found in cases of thrombocytopenia stimulating megakaryopoiesis, but high levels are also seen in hypoxic circumstances and maternal conditions associated with placental insufficiency. Studies report increased TPO levels in the serum of asphyxiated neonates within 48 to 72 h following major stress and hypoxia (30).

In addition, the increased number of NRBC's in the blood smear suggests chronic hypoxia. Under normal conditions, these are not seen in large quantities. Hypoxia is the major stimulus of erythropoietin synthesis resulting in premature release of immature erythrocyte precursors in an attempt to maximize tissue oxygen delivery (31). A study by Christensen et al. claims that it takes at least 28–29 h from the onset of fetal hypoxia before an increase in nucleated red blood cells in the peripheral blood can be detected (32). This corresponds to our hypothesis that the IVH in our case occurred at least several days earlier. Thirdly, neonatal thrombocytopenia is also associated with chronic hypoxia, probably due to secondary megakaryocyte deficiency (31, 33). Thus, we assume that the thrombocytopenia in our case was probably the consequence of chronic hypoxia, rather than the consequence of the IVH. Finally, histopathology of the placenta showed extensive infarction, shown to be more frequent in APS (like syndrome) (34). This has led to impaired uteroplacental vascular function and intra-uterine growth restriction, supportive of chronic hypoxia in the fetus. Unfortunately, our hypothesis could not be strengthened by autopsy, as parents refused this, we consider this a limitation. However, we thoroughly ruled out other etiologies, concluding to OAPS as a diagnosis “by exclusionum.” Supportive is the fact that her subsequent pregnancy was uneventful after pharmacological treatment of OAPS.

We think that the fetal IVH in our case has resulted from chronic hypoxia and inflammation due to OAPS, which, in combination with the lack of cerebral autoregulation at this gestational age, resulted in rupture of the fragile capillary bed of the germinal matrix (24).

## Conclusion

This case demonstrates that OAPS can result in severe neonatal morbidity and mortality. Therefore, we feel that women with early and severe preeclampsia and/or a history of pregnancy loss should be evaluated for OAPS and carefully monitored throughout pregnancy. Further, we advise to test mothers for OAPS in the case of idiopathic fetal hemorrhage.

## Data Availability Statement

The original contributions presented in the study are included in the article/supplementary material, further inquiries can be directed to the corresponding author/s.

## Ethics Statement

The study was reviewed by the Medical Ethics Committee Isala Zwolle The Netherlands. Ethical approval was not required for the study on human participants in accordance with the local legislation and institutional requirements. The patients/participants legal guardian/next of kin provided written informed consent for the publication of any potentially identifiable images or data included in this article.

## Author Contributions

MB wrote the initial manuscript, led revisions, and submitted the manuscript. GW-M critically revised the manuscript for important intellectual content. SN provided obstetric care to the mother and reviewed the manuscript. JB treats the mother and reviewed the manuscript. All authors read and approved the final version of the manuscript.

## Conflict of Interest

The authors declare that the research was conducted in the absence of any commercial or financial relationships that could be construed as a potential conflict of interest.
